# The Epidemiological Characteristics of Stroke in Hunan Province, China

**DOI:** 10.3389/fneur.2018.00583

**Published:** 2018-07-19

**Authors:** Wei He, Yunhai Liu, Jie Feng, Qing Huang, Ji Xu, Xiaojuan Liu, Cheng Yu, Wenbin Zhu, Te Wang, Donghui Jin, Huilin Liu, Yuelong Huang, Biyun Chen

**Affiliations:** ^1^Department of Neurology, Xiangya Hospital, Central South University, Changsha, China; ^2^Hunan Clinical Research Center for Cerebrovascular Disease, Changsha, China; ^3^Department of Anesthesiology and Perioperative Medicine, Oregon Health and Science University, Portland, OR, United States; ^4^Department of Neurology, Changsha Central Hospital, Changsha, China; ^5^Hunan Provincial Center for Disease Control and Prevention, Changsha, China

**Keywords:** prevalence, incidence, morality, stroke, epidemiology, Hunan province

## Abstract

Previous studies have shown that Hunan Province has a high incidence of stroke and a high proportion of intracerebral hemorrhage (ICH). Considering the changes over the past three decades, little is known about the current epidemiological characteristics of stroke in Hunan Province. In 2013, a cross-sectional study was conducted at seven national disease surveillance points (DSPs) in Hunan Province. A multistage cluster sampling method was used to select a representative sample. A total of 21,156 participants aged 20 years and older were examined. Among the 21,156 participants, the number of prevalent strokes, incident strokes and deaths was 307, 87, and 36, respectively. The 2010 China census-standardized prevalence, incidence and mortality were 1191.0 per 100,000 people [95% confidence interval (CI) 1044.8–1337.2], 333.6 per 100,000 person-years (95% CI 255.7–411.5) and 129.7 per 100,000 person-years (95% CI 81.1–178.3), respectively. Ischemic stroke (IS), ICH, subarachnoid hemorrhage (SAH), and stroke of undetermined type (UND) constituted 50.6, 41.4, 5.7, and 2.3% of all incident stroke cases, respectively. Tianxin, Liuyang, Wuling, and Hongjiang have high proportions of ICH (61.5, 58.3, 60, and 50%, respectively). Hypertension is the most common risk factor for prevalent stroke (71.34%), followed by smoking (30.62%) and alcohol use (25.73%). In conclusion, Hunan Province has an extremely heavy stroke burden. The high proportion of ICH is not limited to the Changsha community; it represents an important issue for all of Hunan Province.

## Introduction

Cerebrovascular disease and ischemic heart disease were the leading causes of years of life lost (YLLs) for both sexes in 123 countries in 2016 (1). The Global Disease Burden Report 2015 (2) estimated that from 1990 to 2015, stroke accounted for 47.3% of total disability-adjusted life-years (DALYs) and 67.3% of deaths caused by all neurological disorders. Countries with low to mid-range scores on the socio-demographic index (SDI) have increased stroke rates, and these rates have the lowest values in the countries with the highest SDI scores.

A report by Yang et al. showed that stroke was the leading cause of death in China in 2010 (3). A number of stroke epidemiological studies have been conducted in China since the 1980s. A large stroke epidemiological survey started in 1986 (4), including 5.79 million population samples from 29 provinces and cities in China, and showed that the incidence of stroke in China was 115.9 per 100,000 person-years, and the prevalence of stroke was 259.86 per 100,000 people in China. The geographic distribution of the incidence of stroke in China displayed a north-south gradient, which was characterized by a significantly higher incidence of stroke in northern China than in southern China (5, 6). However, Hunan Province was an exception. Hunan Province is located in South Central China, with a total area of 211,800 square kilometers, and the total population of Hunan Province is 65.68 million. Although this province is located in southern China, the incidence of stroke in Hunan was 141.2 per 100,000 person-years, and the mortality rate reached 86.2 per 100,000 person-years. Another survey that reported 10 consecutive years of stroke incidence monitoring in Beijing, Shanghai and Changsha (the capital of Hunan Province) between 1991 and 2000 showed that the incidence of stroke in Changsha was 150 per 100,000 person-years, which ranked first among the three cities (7). In addition, intracerebral hemorrhage (ICH) accounted from 50.3 to 55.4% of all strokes in Hunan, although it is extremely rare worldwide (8, 9).

Since the beginning of the twenty-first century, there have been some unfavorable changes in the lives of Chinese residents: unhealthy diet structures and lifestyle habits, inevitable aging of the population, urbanization, and other behavioral and social factors (3). Disease patterns have also changed accordingly. On the other hand, previous epidemiological data were mainly from the urban communities in Changsha and did not cover the rural regions. Whether the high proportion of ICH is a phenomenon unique to Changsha or a common characteristic in Hunan Province remains unknown. It is necessary to carry out a new round of epidemiological surveys to further elucidate the stroke burden and the subtypes of stroke in Hunan Province.

## Materials and methods

### Study participants and design

**Figure 1:**
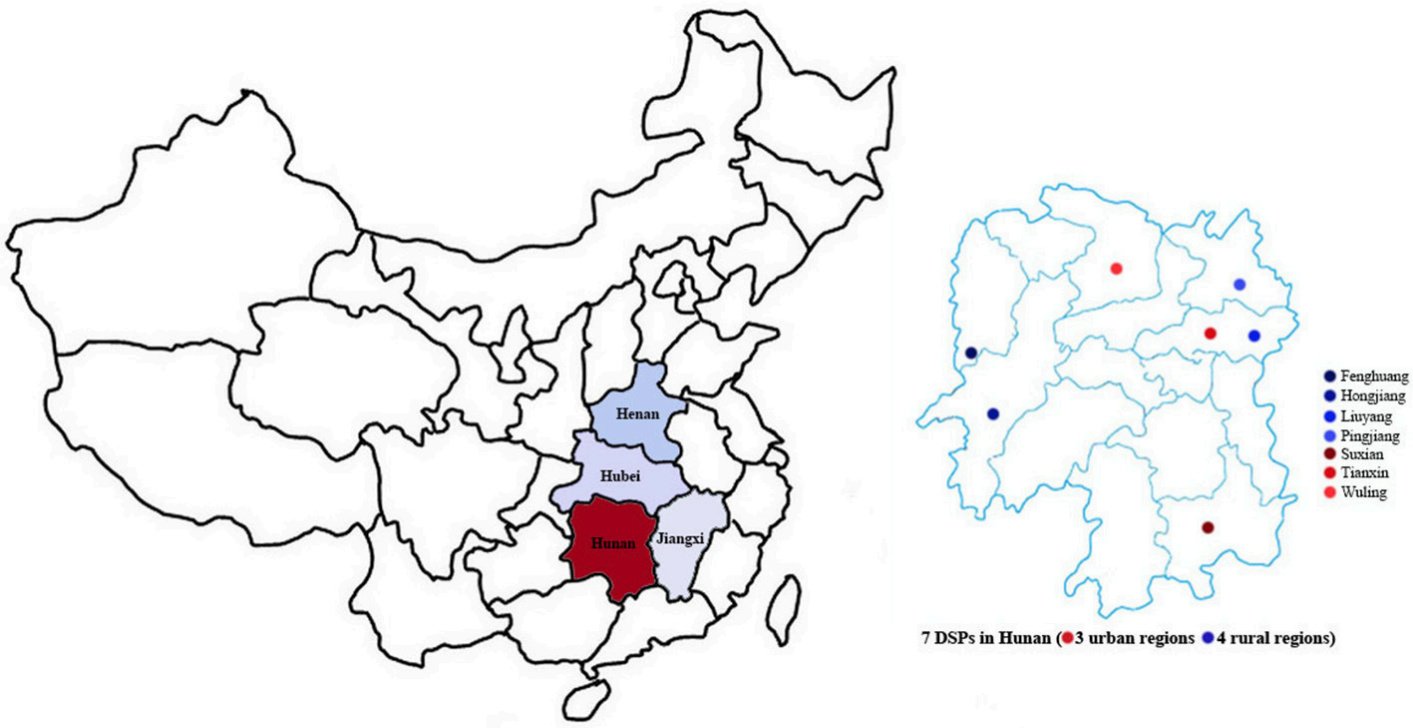
Central China (including Hunan, Jiangxi, Hubei, and Henan) and 7 DSPs in Hunan Province (3 urban regions and 4 rural regions).

The method of this study is consistent with the national epidemiological survey of stroke (10). Our survey was conducted at seven monitoring sites (three urban and four rural regions) (Figure 1), based on the national disease surveillance points (DSPs) system. The survey districts in large, medium, and small cities were defined as urban regions, and other survey districts were defined as rural regions. The sample size of Hunan Province was calculated by a multistage stratified cluster sampling method that accounted for national representation in terms of socio-economic status, geographical distribution, educational level, medical care, and lifestyles. A town/district proportional to the population size of the survey district was selected. In each town/district, cluster sampling was used to select no <4,500 residents (calculated as an average of three persons per household multiplied by ~1,500 households), and at least 85% of those individuals were expected to complete the entire survey process (~3,800 residents from 1,300 households). The actual sample size was at least 26,600 residents for all age groups.

The face-to-face questionnaire surveys were performed door-to-door by Center for Disease Control (CDC) investigators who had been well trained. The study participants were people who had lived in that community (township or street) for at least 6 months. The investigators collected the following information: basic information about each family member (family households are the basic survey units, and all resident populations are included, including non-family members who have lived with the family for at least 6 months, such as nannies, drivers, or other lodged populations), the symptoms and medical history of each individual, and family members who died from stroke or prevalent stroke cases between September 1, 2012, and August 31, 2013. The point in time for determining prevalence was defined as 24:00 August 31, 2013. The incidence of stroke was defined as the rate of first-ever stroke cases that occurred during the year prior to the prevalence point (between 00:00 September 1, 2012, and 24:00 August 31, 2013). All deaths that occurred from 00:00 September 1, 2012, to 24:00 August 31, 2013, were recorded to determine later if stroke was the possible cause of death with a validated verbal autopsy technique (11, 12).

**Figure 2:**
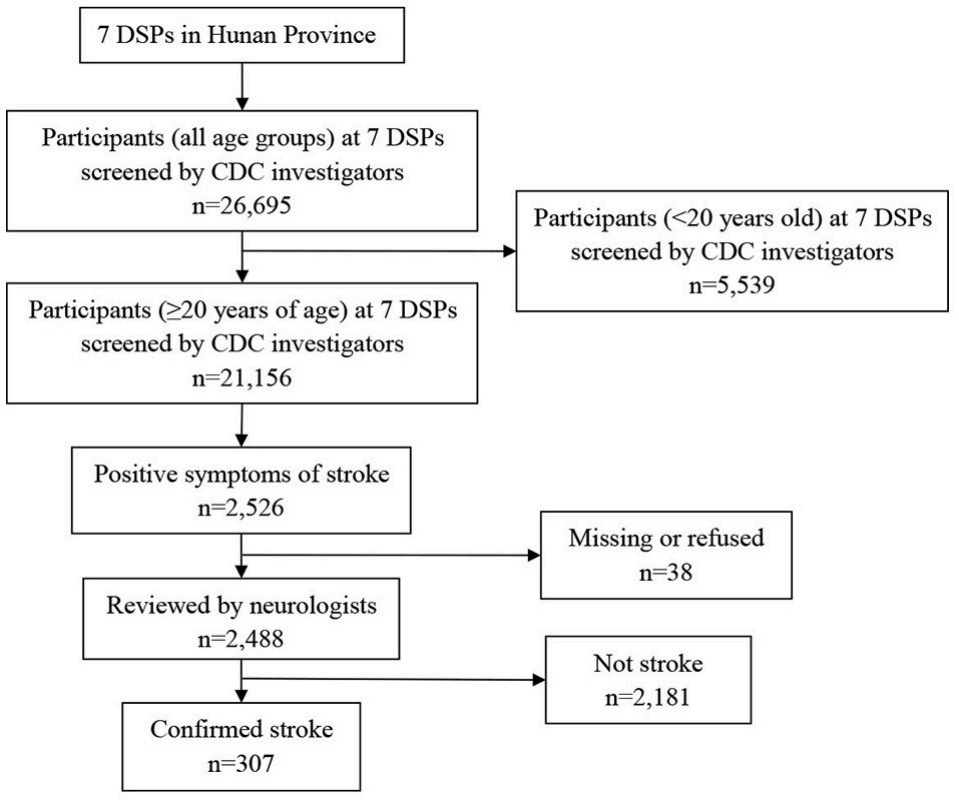
Flowchart of stroke case evaluation.

In the second stage, two neurologists interviewed 2,526 participants with suspected stroke/transient ischemic attack (TIA, including all definite and possible cases) and completed the case confirmation forms. There were 38 patients who declined the interview or follow-up. First-ever stroke cases occurring between September 1, 2012, and August 31, 2013, were identified as incident strokes. All living subjects with confirmed stroke before August 31, 2013, were identified as prevalent stroke cases. People who died from stroke from September 1, 2012, to August 31, 2013, were used to calculate the mortality rate of stroke. All case reviews were supported by medical records, image data, official statistics, and death certificates (for fatal events). When appropriate, some study participants were requested to undergo a brain neuroimaging examination (for example, to exclude brain disorders mimicking stroke, such as hypertensive encephalopathy, infection, toxic/metabolic encephalopathy, Wernicke encephalopathy, epileptic seizure, mitochondrial encephalopathy, and transient global amnesia) and/or another neurological examination (lumbar puncture or electroencephalogram) (Figure 2).

### Diagnostic criteria

The diagnostic criteria for stroke were based on the World Health Organization (WHO) criteria (13): rapidly developing clinical signs of focal (or global) disturbance of cerebral function, lasting more than 24 h or leading to death, with no apparent cause other than that of vascular origin. The exclusion criteria were other nervous system abnormalities induced by trauma, metabolic disorders, tumors, or central nervous system infections. The pathological type of stroke was classified into four major categories: (1) subarachnoid hemorrhage (SAH) (lumbar puncture was the only diagnostic method for SAH); (2) ICH; (3) ischemic stroke (IS); and (4) undetermined type (UND). Stroke cases for which no brain imaging was performed within the first week of stroke onset or for which the results of imaging or autopsy could not be obtained for further confirmation by two neurologists were classified as UND.

### Data collection

The research staff reviewed all questionnaires that were sent to the Beijing Neurosurgical Institute prior to the end of 2013. Then, all questionnaires were entered into a database with a standard procedure. All research staff received standardized training and were formally certified before data collection. The quality and completeness of the questionnaires from each investigation site were verified and monitored by a professional quality controller to ensure compliance with the standardized study protocol. A strict double-entry system was used for quality control during the data collection and cleanup process. The Clinical Research Organization surveyed and monitored the entire survey process to ensure the consistency of data collection among all study sites. The study was approved by the ethical review committees of Beijing Tiantan Hospital and Xiangya Hospital. All interviewers obtained written informed consent before data collection.

Education, marital status, current occupation, current smoking (≥1 cigarette/per day) and alcohol intake (any dose of alcohol, ≥1 time per week) were defined by the participants' self-reports. A history of hypertension was defined as documented systolic blood pressure ≥140 mmHg or diastolic blood pressure ≥90 mmHg or the use of blood pressure-lowering drugs. A history of diabetes mellitus was defined as documented fasting blood glucose ≥7.0 mmol/L and/or 2-h postprandial blood glucose ≥11.1 mmol/L or the use of antidiabetic drugs. Confirmed medical records of atrial fibrillation were diagnosed by electrocardiograph (ECG). Documented medical records of myocardial infarction, angina or hyperlipidemia were used to diagnose coronary heart disease (CHD) or dyslipidemia. To identify differences in the prevalence of risk factors between the two groups, all the risk factors were classified as Yes or No and analyzed as binary variables. The missing data were classified as No because we cannot accurately estimate the prevalence of all risk factors in epidemiological surveys.

### Statistical analysis

Age- and sex-specific prevalence (per 100,000 people), incidence, and mortality rates (per 100,000 person-years) were estimated. Age-standardized prevalence, incidence and mortality rates were calculated with a direct method by using the 2010 China population census as standard, 95% confidence intervals (CIs) and the relative ratio (RR) of 10 years age group of the prevalence, incidence, and mortality of stroke were calculated by using the Poisson distribution. The chi-squared (χ^2^) test was used to assess differences in the prevalence, incidence and mortality of stroke between sex, the prevalence of risk factors for prevalent stroke between sexes and between rural and urban populations. All statistical analyses were conducted in SPSS 22.0 (SPSS Inc., Chicago, IL, USA).

## Results

The characteristics of the study participants are shown in Table 1. The response rate was 85.9%. The mean age was 46.2 (SD 16.8) years. An educational background of primary school or less accounted for 37% of participants; people who were married or had a partner accounted for 79.4% of participants; farmers accounted for 67.5% of participants; and urban residents accounted for 42.6% of participants.

**Table 1:** Characteristics of the 21,156 participants (≥20 years) in 2013.

**Characteristics**	**Overall**	**Men**	**Women**	***p***
Participants, *n* (%)	21,156 (100.0)	10,566 (49.9)	10,590 (50.1)	
Residence, *n* (%)				<0.001
Urban	9,009 (42.6)	4,347 (41.1)	4,662 (44.0)	
Rural	12,147 (57.4)	6,219 (58.9)	5,928 (56.0)	
Age groups, *n* (%)				<0.001
20–29	4,470 (21.1)	2,072 (19.6)	2,398 (22.6)	
30–39	3,556 (16.8)	1,822 (17.2)	1,734 (16.4)	
40–49	4,819 (22.8)	2,493 (23.6)	2,326 (22.0)	
50–59	3,292 (15.6)	1,700 (16.1)	1,592 (15.0)	
60–69	2,816 (13.3)	1,423 (13.5)	1,393 (13.2)	
70–79	1,590 (7.5)	783 (7.4)	807 (7.6)	
≥80	613 (2.9)	273 (2.6)	340 (3.2)	
Education, *n* (%)				<0.001
Primary school or lower	7,829 (37.0)	3,646 (34.5)	4,183 (39.5)	
Middle school	11,776 (55.7)	6,136 (58.1)	5,640 (53.3)	
College and higher	1,146 (5.4)	581 (5.5)	565 (5.3)	
Missing	405 (1.9)	203 (1.9)	202 (1.9)	
Marital status, *n* (%)				<0.001
Married	16,788 (79.4)	8,322 (78.8)	8,466 (79.9)	
Single	2,409 (11.4)	1,452 (13.7)	957 (9.0)	
Widowed	1,549 (7.3)	578 (5.5)	971 (9.2)	
Missing	410 (1.9)	214 (2.0)	196 (1.9)	
Occupation, *n* (%)				<0.001
Student	292 (1.4)	111 (1.1)	181 (1.7)	
Worker	217 (1.0)	145 (1.4)	72 (0.7)	
Farmer	14,281 (67.5)	7,394 (70.0)	6,887 (65.0)	
Employee	1,307 (6.2)	777 (7.4)	530 (5.0)	
Entrepreneur	2,793 (13.2)	1,537 (14.5)	1,256 (11.9)	
Retired or unemployed	1,727 (8.2)	312 (3.0)	1,415 (13.4)	
Missing	539 (2.5)	290 (2.6)	249 (2.3)	

*The term “workers” refers to individuals who work at factories. “Employees” refers to national public servants, professional technicians, office clerks, business managers, and active servicemen. “Entrepreneurs” refers to individual operators and freelancers*.

In this study, the diagnostic rate of computed tomography (CT) and/or magnetic resonance imaging (MRI) in stroke patients was 93.1% among incident strokes and 87.9% among prevalent strokes. Of the 21,156 total participants, the number of prevalent strokes was 307 (1451.1 per 100,000), and the 2010 China population census-standardized prevalence was 1191.0 per 100,000 people. The crude prevalence of stroke was 1476.4 per 100,000 people for men and 1425.9 per 100,000 people for women (Table 2). The 2010 China population census-standardized prevalence for men was slightly higher than that observed for women (1222.1/100,000 and 1154.0/100,000, respectively). A study of risk factors in 307 people with prevalent stroke showed that hypertension was the most common risk factor (71.34%), followed by smoking (30.62%) and alcohol use (25.73%) (Table 5). There were significant differences in smoking between men and women (*p* < 0.001) and in alcohol use between urban and rural subjects (*p* < 0.01). There were no significant differences in hypertension, diabetes, dyslipidemia, arterial fibrillation, and CHD between men and women or between urban and rural subjects.

**Table 2:** Prevalence (with 95% CIs) of stroke per 100,000 Hunan adults by sex in 2013.

**Age group (years)**	**Men**			**Women**			**Total**		
	**No. of strokes**	**Prevalence**	**95% CI**	**No. of strokes**	**Prevalence**	**95% CI**	**No. of strokes**	**Prevalence**	**95% CI**
20–29	1	48.3	0–142.8	0	0	0–0	1	22.4	0–66.2
30–39	2	109.8	0–261.8	1	57.7	0–170.7	3	84.4	0–180.0
40–49	8	320.9	98.9–542.9	9	386.9	134.6–639.2	17	352.8	185.4–520.2
50–59	38	2,235.3	1,532.6–2,938.0	30	1,884.4	1,216.5–2,552.4	68	2,065.6	1,579.7–2,551.5
60–69	54	3,794.8	2,802.0–4,787.6	44	3,158.7	2,240.2–4,077.1	98	3,480.1	2,803.2–4,157.0
70–79	37	4,725.4	3,239.2–6,211.6	41	5,080.5	3,565.4–6,595.7	78	4,905.7	3,844.0–5,967.3
≥80	16	5,860.8	3,074.4–8,647.2	26	7,647.1	4,822.2–10,471.9	42	6,851.6	4,851.7–8,851.4
Total	156	1,476.4	1,246.5–1,706.4	151	1,425.9	1,200.1–1,651.7	307	1,451.1	1,290.0–1,612.3
ASR^*^		1,222.1	1,012.6–1,431.6		1,154.0	950.6–1,357.4		1,191.0	1,044.8–1,337.2

* *ASR, Age-standardized rates based on the 2010 China population census*.

The number of incident strokes was 87, and the crude incidence of stroke in Hunan Province was 413.3 per 100,000 person-years; the 2010 China population census-standardized incidence rate was 333.6 per 100,000 person-years. The crude incidence of stroke was 447.0 per 100,000 people for men and 379.6 per 100,000 people for women (Table 3). The 2010 China population census-standardized incidence for men was slightly higher than that observed for women (367.3/100,000 vs. 287.2/100,000, respectively).

**Table 3:** Incidence of stroke per 100,000 person-years (with 95% CIs) among Hunan adults (≥20 years) by sex in 2012–2013.

**Age group (years)**	**Men**			**Women**			**Total**		
	**No. of strokes**	**Rate**	**95% CI**	**No. of strokes**	**Rate**	**95% CI**	**No. of strokes**	**Rate**	**95% CI**
20–29	1	48.3	0–142.8	0	0	0–0	1	22.4	0–66.2
30–39	1	54.9	0–162.6	0	0	0–0	1	28.1	0–83.3
40–49	3	120.4	0–256.6	0	0	0–0	3	62.3	0–132.8
50–59	9	531.0	185.0–877.0	4	251.6	5.3–497.8	13	395.7	181.0–610.4
60–69	16	1,131.5	580.2–1,682.9	15	1,083.0	537.9–1,628.1	31	1,107.5	719.8–1,495.3
70–79	8	1,051.2	326.6–1,775.9	7	885.0	232.3–1,537.6	15	966.5	479.8–1,453.2
≥80	9	3,448.3	1,234.6–5,662.0	14	4,375.0	2,133.9–6,616.1	23	3,958.7	2,373.2–5,544.2
Total	47	447.0	319.5–574.5	40	379.6	262.2–497.0	87	413.3	326.6–499.9
ASR^*^		376.3	259.3–493.3		287.2	185.0–389.4		333.6	255.7–411.5

* *ASR, Age-standardized rates based on the 2010 China population census*.

The number of deaths due to stroke was 36. The mortality rate of stroke was 170.6 per 100,000 person-years, and the 2010 China population census-standardized mortality was 129.7 per 100,000 person-years. The mortality of stroke was 161.7 per 100,000 person-years for men and 179.4 per 100,000 person-years for women. The 2010 China population census-standardized mortality for men was slightly higher than that observed for women (130.5/100,000 and 125.5/100,000) (Table 4).

**Table 4:** Mortality (with 95% CIs) of stroke per 100,000 Hunan adults (≥20 years) by sex in 2012–2013.

**Age group (years)**	**Men**			**Women**			**Total**		
	**No. of strokes**	**Rate**	**95% CI**	**No. of strokes**	**Rate**	**95% CI**	**No. of strokes**	**Rate**	**95% CI**
20–29	0	0	0–0	0	0	0–0	0	0	0–0
30–39	0	0	0–0	0	0	0–0	0	0	0–0
40–49	1	40.1	0–118.8	0	0	0–0	1	20.8	0–61.5
50–59	1	59.0	0–174.6	1	62.9	0–186.0	2	60.9	0–145.2
60–69	4	282.7	6.0–559.3	4	287.2	6.1–568.2	8	284.9	87.8–482.0
70–79	6	789.5	160.3–1,418.7	3	373.1	0–794.6	9	575.4	200.6–950.3
≥80	5	1,915.7	252.7–3,578.7	11	3,188.4	1,334.5–5,042.4	16	2,640.3	1,363.7–3,916.8
Total	17	161.7	84.9–238.5	19	179.4	98.8–260.0	36	170.6	114.9–226.3
ASR^*^		130.5	61.5–199.5		125.5	57.9–193.1		129.7	81.1–178.3

* *ASR, Age-standardized rates based on the 2010 China population census*.

**Table 5:** Prevalence of risk factors among 307 people with prevalent stroke by sex and residency in Hunan.

		**Sex**			**Urban and Rural**			
		**Men *n* (%)**	**Women *n* (%)**	***p***	**Urban *n* (%)**	**Rural *n* (%)**	**Total *n* (%)**	***p***
Hypertension	Yes	113 (72.44%)	106 (70.20%)	0.665	98 (71.01%)	121 (71.60%)	219 (71.34%)	0.911
	No	43 (27.56%)	45 (29.80%)		40 (28.99%)	48 (28.40%)	88 (28.66%)	
Diabetes	Yes	11 (7.05%)	16 (10.60%)	0.273	13 (9.42%)	14 (8.28%)	27 (8.79%)	0.715
	No	145 (92.95%)	135 (89.40%)		125 (90.58%)	156 (91.72%)	280 (91.21%)	
Dyslipidemia	Yes	29 (18.59%)	25 (16.56%)	0.640	27 (19.57%)	27 (15.98%)	54 (17.59%)	0.411
	No	127 (81.41%)	126 (83.44%)		111 (80.43%)	142 (84.02%)	253 (82.41%)	
Atrial fibrillation	Yes	4 (2.56%)	3 (1.99%)	1.000	3 (2.17%)	4 (2.37%)	7 (2.28%)	1.000
	No	152 (97.44%)	148 (98.01%)		135 (97.83%)	165 (97.63%)	300 (97.72%)	
CHD	Yes	22 (14.10%)	30 (19.87%)	0.178	23 (16.67%)	29 (17.16%)	52 (16.94%)	0.909
	No	134 (85.90%)	121 (80.13%)		115 (83.33%)	140 (82.84%)	255 (83.06%)	
Current smoker	Yes	62 (39.74%)	32 (21.19%)	<0.001	49 (35.51%)	45 (26.63%)	94 (30.62%)	0.093
	No	94 (60.26%)	119 (78.81%)		89 (64.49%)	124 (73.37%)	213 (69.38%)	
Alcohol use	Yes	44 (28.21%)	35 (23.18%)	0.314	46 (33.33%)	33 (19.53%)	79 (25.73%)	< 0.01
	No	112 (71.79%)	116 (76.82%)		92 (66.67%)	136 (80.47%)	228 (74.27%)	

**Figure 3:**
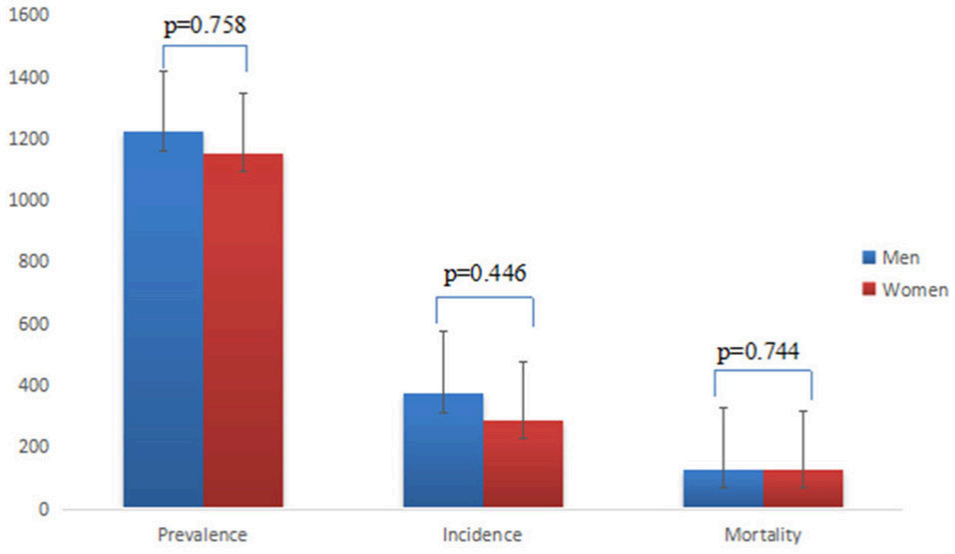
Sex-specific prevalence, incidence, and mortality.

**Figure 4:**
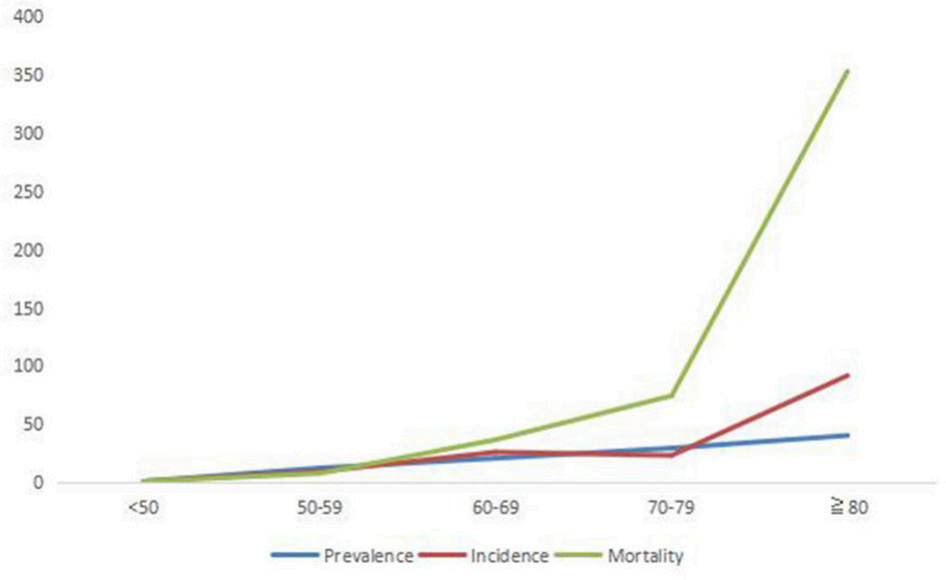
The RR of 10 years age group of the incidence, prevalence, and mortality of stroke.

There is no significant difference between sex-specific prevalence, incidence and mortality (*p* > 0.05) (Figure 3). With reference to the age <50 years, the RR of 10 years age group of incidence, prevalence and mortality of stroke are increasing, especially for the RR of mortality of stroke (Figure 4).

IS, ICH, SAH, and UND constituted 50.6, 41.4, 5.7, and 2.3% of all incident strokes, respectively. Tianxin, Liuyang, Wuling, and Hongjiang had high proportions of ICH (61.5, 58.3, 60, and 50%, respectively) (Table 6).

**Table 6:** Number and percentage of incident strokes observed in Hunan Province by stroke subtype.

	**Strokes**	**IS (%)**	**ICH (%)**	**SAH (%)**	**UND (%)**
Tianxin	13	2 (15.4%)	8 (61.5%)	3 (23.1%)	0
Liuyang	12	4 (33.3%)	7 (58.3%)	1 (8.3%)	0
Pingjiang	14	8 (57.1%)	4 (28.6%)	0	2 (14.3%)
Fenghuang	10	7 (70%)	3 (30%)	0	0
Wuling	5	2 (40%)	3 (60%)	0	0
Hongjiang	12	5 (41.7%)	6 (50%)	1 (8.3%)	0
Suxian	21	16 (76.2%)	5 (23.8%)	0	0
Total	87	44 (50.6%)	36 (41.4%)	5 (5.7%)	2 (2.3%)

*IS, ischemic stroke; ICH, intracerebral hemorrhage; SAH, subarachnoid hemorrhage; UND, stroke of undetermined pathological type*.

## Discussion

Our study shows that the prevalence, incidence, and mortality of stroke in Hunan Province are much higher than those reported in the survey performed in 1986 (1165.3 vs. 259.86, 324.7 vs. 115.87, and 117.6 vs. 80.94, respectively) (age-standardized rates based on the world standard population) (4, 5). According to the data from the Sixth National Population Census, the total population of Hunan Province is 65.68 million, and the number of people suffering from stroke in Hunan Province is ~765,000. The synchronous national stroke survey showed that the prevalence of stroke in central China (including Hunan, Jiangxi, Hubei, and Henan) was the highest among the seven regions of China, and the incidence and mortality of stroke in central China ranked second among these regions (10). The incidence of stroke in Hunan Province is close to the incidence in central China (Hunan/Central China 333.6/326.1), and the mortality of stroke in Hunan Province is lower than that in central China (Hunan/Central China 129.7/158.5), which indicates that stroke in Hunan Province is a very serious burden, yet the risk is still increasing. On the other hand, the incidences of stroke are significantly higher in some high-latitude countries than in low-latitude countries (14, 15). Our study confirmed that the stroke incidence of Hunan Province does not follow a north-south gradient, which has been supported by previous studies (7, 16, 17). Environmental or climatic factors may account for a portion of the high incidence of stroke in Hunan Province.

Our data show that hypertension is the most common risk factor in patients with prevalent stroke (71.34%), but no significant differences were found between sexes or between rural and urban areas (*p* > 0.05). Previous studies have also shown that hypertension is highly correlated with stroke incidence and mortality and explains 70% of the geographic variability in stroke incidence and mortality in China (5, 18). Other researchers have found that the prevalence of hypertension among patients with prevalent stroke in China is higher than that in other countries (19). Similarly, Gu (20) showed that the age-specific prevalence of hypertension in men was higher than that in women in the population aged 35 to 64 years but not in the population aged 65–74 years. However, our data were obtained mainly from oral inquiries, and missing data were classified as not having the corresponding disease (No), which may lead to underestimated rates of risk factors. Smoking and alcohol use have been identified as common risk factors for stroke (21, 22). The proportion of smoking in men is higher than that in women (*p* < 0.01), which may partially explain the higher prevalence of stroke in men than in women. The reason why alcohol consumption is more frequent in urban areas than in rural areas may be related to an unhealthy urban lifestyle in Hunan (such as drinking alcohol and eating late at night) (*p* < 0.01). The Global Burden of Disease Study 2013 (GBD 2013) stated that over 92% of the stroke burden is due to modifiable risk factors, with behavioral factors (i.e., smoking) accounting for 74.2% and metabolic factors (i.e., high systolic blood pressure) accounting for 72.4% of the stroke burden (23). Therefore, controlling risk factors for stroke is important for decreasing the stroke burden.

The main subtypes of incident stroke in Hunan Province are IS and ICH, which account for 50.6 and 41.4% of all strokes, respectively. From a nationwide perspective, IS is still the main subtype and accounts for 45.5–75.9% of strokes, whereas ICH accounts for 17.1–55.4% of strokes (8, 24, 25). In western countries, the proportions of IS and ICH are 67.3–80.5 and 6.5–19.6%, respectively (26). Changsha is the capital of Hunan Province, and it has been reported as the city with the highest prevalence of ICH among the three cities (Changsha, Beijing and Shanghai) (7). A study by Yang et al. (8) showed that ICH accounted for 55.4% of strokes in the Changsha community between 1986 and 2000. In a study by Sun et al. (9), the incidence of IS increased at an annual rate of 3.5%, while the incidence of ICH exhibited no significant changes. This new round of stroke epidemiological studies showed that not only Changsha community (Tianxin) but also other regions of Hunan Province [one urban region (Wuling) and two rural regions (Liuyang and Hongjiang)] have a high proportion of ICH. Hypertension is the most important risk factor for ICH (27, 28), and hypertensive patients are increasing (29). We also found that hypertension is the most common risk factor in patients with prevalent stroke (71.34%). Low awareness, inadequate treatment and an uncontrolled rate of hypertension may explain the high proportion of ICH. According to the 2002 National Nutrition and Health Survey (NNHS), the awareness, treatment and control of hypertension were achieved in only 30%, 25 and 6% of participants, respectively (30). However, not all of the seven investigated regions had a high proportion of ICH. Low awareness and poor control of the rate of hypertension cannot fully explain this phenomenon. Hunan residents tend to eat a spicy and salty diet and to adopt an unhealthy lifestyle of drinking alcohol and eating late at night, and there are also regional differences among these seven regions, which represent potential risk factors for ICH and hypertension. Furthermore, a study by Woo et al. (31) investigated the genetic and environmental risk factors for ICH and found that a third of lobar ICH cases were associated with the apolipoprotein E4 or E2 allele. The fundamental reason for the high proportion of ICH remains unclear, although it may result from a combination of hypertension, diet, lifestyle, and genetic and environmental factors. Further research with a larger sample size should be conducted to investigate blood pressure management status, dietary habits, lifestyle, and genetic risk factors.

## Limitations of the present survey

There are several potential limitations of the present epidemiological survey. First, the risk factor data were obtained from the oral responses of the investigators and were classified as either Yes or No and analyzed as binary variables, which may have incurred bias. Second, recall bias may have affected the evaluation of the prevalence, incidence, and mortality of stroke. However, great efforts have been made to minimize these possibilities by cross-checking the data obtained from the door-to-door interviews and from medical records and by asking neurologists to review the identified and suspected cases. Third, we used only smoking cigarettes as the dominant mode of tobacco use in our survey; this approach may underestimate the proportion of tobacco intake. Fourth, the use of a small monitoring sample (average 3,700 residents per DSP) in a single DSP and the limited medical conditions in some rural areas led to an imbalanced proportion of stroke subtypes, such as a higher proportion of SAH than IS in Tianxin; in addition, the proportion of UND in Pingjiang reached 14.3%, and the number of incident strokes in Wuling was only 5, which led to a 60% proportion of ICH in Wuling.

## Conclusion

In conclusion, Hunan Province has an extremely heavy stroke burden. The high proportion of ICH is not limited to the Changsha community; it represents an important issue for all of Hunan Province.

## Author contributions

YL and YH involved in the study design. DJ, HL, BC were responsible for the first stage data collection. JF, WH, JX, TW, XL, QH, and CY were responsible for the second stage cases confirmation. WH wrote the manuscript. YL and WZ modified and revised the manuscript. All authors have read and approved the final version of the manuscript.

### Conflict of interest statement

The authors declare that the research was conducted in the absence of any commercial or financial relationships that could be construed as a potential conflict of interest.
